# Effect of adapted dance program on gait in adults with cerebral palsy: a pilot study

**DOI:** 10.3389/fneur.2024.1443400

**Published:** 2024-10-14

**Authors:** Hee Joung Joung, Tae Hoon Kim, Moon Seok Park

**Affiliations:** ^1^Sport Science Laboratory, Changwon National University, Gyeongnam, Republic of Korea; ^2^Department of Dance, Changwon National University, Gyeongnam, Republic of Korea; ^3^Department of Orthopedic Surgery, Seoul National University Bundang Hospital, Gyeonggi, Republic of Korea; ^4^College of Medicine, Seoul National University, Seoul, Republic of Korea

**Keywords:** cerebral palsy, dance, ballet, gait, Gait Deviation Index

## Abstract

**Background:**

The gait function in adults with cerebral palsy (CP) deteriorates rapidly with age. Dance has been used as an effective intervention to improve balance, postural control, and gait. This study aimed to investigate the feasibility and effects of an adapted dance program (ADP) on the gait in adults with CP. The ADP, which consists of floor and barre workouts, was designed to be adapted for individuals with CP.

**Method:**

Ten female adults with spastic diplegic CP (mean age 52.3 ± 6.34, Gross Motor Function Classification System level II) participated in this study. Outcome measures, examined using 3D gait analysis, included spatiotemporal gait parameters and the Gait Deviation Index (GDI) based on nine kinematic variables in all planes of motion. To assess feasibility, we conducted post-questionnaires and a group interview. The ADP, each lasting 90 min, was held twice per week for 12 weeks.

**Results:**

A statistically significant improvement was observed in GDI (*Δ*5.74 points, *p* = 0.014), with a large effect size (*d* = 0.76). Foot off (Δ-0.72%), first double support (Δ-0.2%), second double support (Δ1.5%), and single support (Δ0.64%) showed no significant differences. Step length (Δ1.48 cm), cadence (Δ3.95 steps/min), and walking speed (Δ6.41 cm/s) tended to increase, though the differences were not statistically significant. Participants expressed high levels of physical and emotional satisfaction, suggesting a need for early intervention.

**Conclusion:**

The ADP may improve gait patterns in adults with spastic diplegic CP. The feasibility results indicated that the ADP is suitable for adults with spastic diplegic CP. This study provides evidence for improvement in gait patterns through dance, which has not been reported in previous dance studies on individuals with CP, offering additional information on the benefits of dance.

## 1 Introduction

Cerebral palsy (CP) is a non-progressive neurodevelopmental disorder, which is characterized by muscle weakness and spasticity, reduced cardiorespiratory endurance, postural instability, and balance impairment (1). Although the condition is non-progressive, functional deteriorations occur over the patient’s lifespan, leading to premature functional decline among adults with CP (2). Moreover, functional decline and secondary medical complications, such as diabetes, hypertension, cardiovascular conditions, joint pain, fatigue, and other musculoskeletal pain, can impact the performance of the activities of daily living, exercise, social activities, and quality of life (1). Gait performance is a key physical function related to independent activities of daily living and social participation. Previous studies have shown that 35% of adults with CP experience a decline in gait function, 9% stop walking altogether, and most face worsening mobility before the age of 35 (3, 4). These findings indicate the need for effective interventions aimed at restoring gait and preventing rapid deterioration of mobility among adults with CP.

Dance is considered an enjoyable and effective intervention for the neurological population (i.e., people with Parkinson’s disease, stroke, multiple sclerosis, dementia and Alzheimer’s disease, and CP), supporting the enhancement of gait, balance, postural control, and cognitive, social, and emotional aspects (5, 6). Dance also offers benefits for people with CP in terms of motor functions, including balance, gait, cardiorespiratory fitness, and postural control (7, 8). In a recent systematic review and meta-analysis on the efficacy of dance interventions in CP (9), the most commonly investigated outcome measures were mobility, postural control, and balance, showing large effect sizes (*g* > 1.5). However, most studies have targeted children and adolescents with CP (9), and few studies have focused on adults. One study involved creative dance [age 30–54 years, Gross Motor Function Classification System (GMFCS) level I-II, *n* = 10] (10), while the other study performed a wheelchair dance (age 37.6–64.6 years, GMFCS level V, *n* = 6) (11). To our knowledge, the evidence regarding dance interventions for adults with CP remains limited compared to that for children and adolescents (7, 9).

This pilot study aimed to examine the effect of the adapted dance program (ADP) on gait in spastic diplegic adults with CP. Additionally, it also aimed to explore the feasibility of population-specific adaptations (12). We hypothesized that the ADP would improve gait in adults with spastic diplegic CP. The gait performance was evaluated using spatiotemporal gait parameters (i.e., foot-off percentage, step length, cadence, and walking speed) and the Gait Deviation Index (GDI), employing a three-dimensional gait analysis (3DGA) rarely utilized in previous dance research literature on people with CP (7, 9). These parameters are commonly used to evaluate gait quality in clinical settings and scientific research, facilitating the detection of gait changes after interventions (13, 14). The GDI, based on nine kinematic data points from the pelvis, hip, knee, ankle, and foot, represents a numerical variable that indicates the quality of the gait pattern. It serves as an index of gait pathology with a range of 0–100. A value greater than 100 indicates the absence of gait pathology (15). This study contributes to the body of evidence regarding the effects of dance on adults with CP and suggests that adaptations are exploratory and intended to inform future studies and practitioners.

## 2 Method

### 2.1 Study design

This pilot study used a non-randomized study design with pre- and post-assessment comparisons to examine the effect of ADP on gait and explore the feasibility of the ADP in adults with spastic diplegic CP, using an exit questionnaire and interview. We expect that this study will provide meaningful information for conducting large-scale studies for potential future use (12, 16). To address the aim of the pilot study, we followed a checklist and guidance entitled TIDieR (Template for Intervention Description and Replication) to improve the completeness of reporting and replicability (16). Because this was a pilot study, a formal calculation of the sample size was not conducted. Instead, the sample size was determined based on a systematic review and meta-analysis of dance interventions for individuals with CP (9) and two intervention studies. In a study by Duarte et al. (9), a sample size of 10–13 participants showed a large effect size (*g* = 1.96). Given that the previous study evaluated gait performance after dance intervention in adults with CP (10) and that our present study shared the same duration and time per class of the intervention, we expect to achieve the same effect size. Nine children with CP were recruited to investigate the effect of ballet use on gait in a pilot study (17). We recruited 12 participants as the target sample, with a dropout rate of 20%. The participants were recruited through advertisements on social networking services targeting the cerebral palsy community in the Republic of Korea between December 2023 and January 2024. Assessments were performed 1 week before (pre-assessment) and 1 week after (post-assessment) the intervention. The intervention was conducted twice a week for 12 weeks, from April to June 2024. After the last intervention, we administered an exit questionnaire and conducted group interviews to examine the feasibility of the intervention. This study was approved by the Institutional Review Board of Seoul National University Bundang Hospital (IRB NO. B-1908-5559-005). All participants signed an informed consent form approved by our hospital, in which the procedure, possible risks, and benefits were described in detail. Informed consent was obtained from all the participants or their legal guardians before the start of the study.

### 2.2 Participants

**Figure 1:**
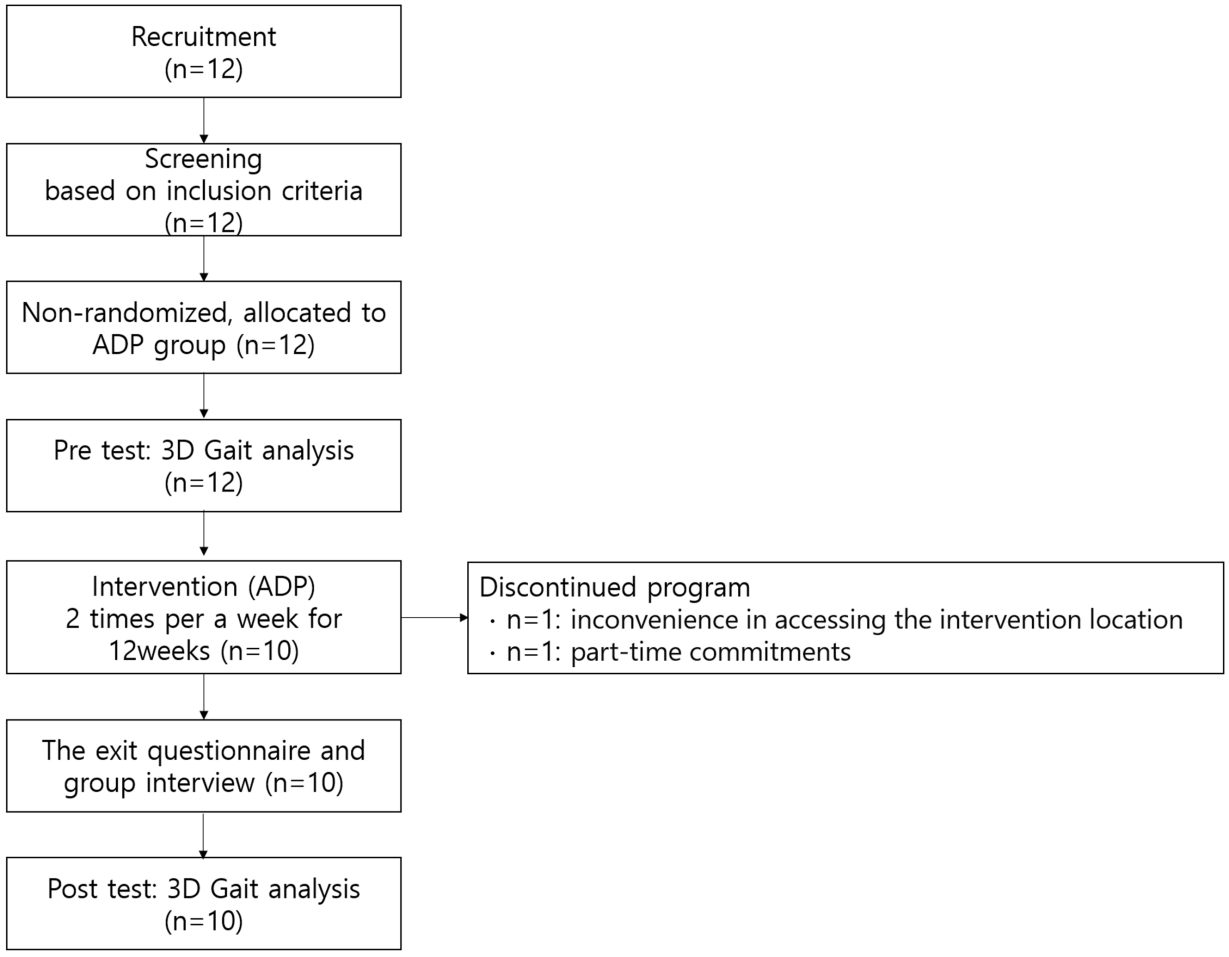
Study flow diagram.

Twelve adults with spastic diplegic CP were recruited voluntarily, and all met the inclusion criteria. The cutoff age was determined by carefully considering the age at which significant changes in walking function are most likely to occur, based on a physical therapists’ perspective (18) that, before the age of 35, the ability to walk in adults with CP declines. The GMFCS is a 5-level classification system that describes gross motor function in children with CP (19). However, it has also proved to be a reliable classification instrument for adults with CP (20). In the context of the Surveillance of Cerebral Palsy in Europe (SCPE) (21), the GMFCS is used to categorize walking ability as follows: GMFCS levels I-II (independent walkers) are classified as “mild,” GMFCS level III (walkers with aid) as “moderate,” and GMFCS levels IV-V (wheelchair users) as “severe.” The participants were recruited according to the following inclusion criteria: (1) diagnosis of spastic diplegic CP confirmed by an orthopedist; (2) male or female age > 30 years; (3) Gross Motor Function Classification System (GMFCS) level I or II (22); (4) ability to walk independently; (5) ability to complete a health screening questionnaire and understand the study protocol; (6) no history of surgery during the past 3 months; and (7) not currently receiving regular physical therapy. The exclusion criteria were as follows: (1) inability to understand the study instructions; (2) medical contraindications to exercise; and (3) use of walking aids. Two participants dropped out during the program because of the inconvenience in accessing the intervention location and their part-time commitments. Ten participants completed the study (Figure 1).

### 2.3 Intervention

The ADP is composed of floor and barre workouts. The program was modified to consider the physical characteristics of individuals with CP, including gait instability, muscle stiffness, postural instability, and coordination difficulties. Thus, the ADP focused on improvements in full-body stretching, range of motion (ROM), balance, postural control, upper and lower limb coordination, and sensory perception, such as musical cues and visual feedback. The participants imitated the instructor’s movements and were encouraged to accompany all the movements with music.

One main instructor and one assistant, both of whom majored in dance education in college, had 16 and 8 years of dance teaching experience for healthy individuals and those with disabilities, respectively. The instructor taught the ADP and demonstrated movement, while the assistant provided posture corrections to maintain proper alignment and offered balance support for safety. The program was conducted in a dance studio equipped with full-length mirrors, ballet bars, yoga mats, and a dance floor. Recorded music was used, and the instructor provided verbal rhythm counts as needed. The ADP lasted 90 min per session, twice a week for 12 weeks, totaling 24 sessions. All sessions were performed as a group. Each session began with a floor-lying workout (30 min), intermission (10 min), barre workout (40 min), and cool-down phase (10 min). Intensity was measured in two sets of four to eight repetitions, taking into account exercise considerations for CP (23). (Refer to the Supplementary material for the detailed movement description in the ADP).

The floor workout was performed in the lying-down position. This modification responds to the features of CP that differ from those of traditional ballet training. The lying down position can release tension or stiffness throughout the body, mitigate unnecessary tension on non-targeted muscles, and compensate for movement. The floor-lying workout comprised parts 1 and 2. Part 1 consists of five movements focused on full-body stretching and increasing ROM: big X full-body stretching, lying feet point and flex, hamstring stretching, hip abduction and adduction, and knee drop. Part 2 consisted of four movements that focused on body perception and coordination between the upper and lower limbs, based on Bartenieff Fundamentals (BF) basic movements (24) and two ballet positions. This included toe tapping, lying first position plié, lying port de bras, and an upper-lower limb connectivity sequence. It is structured to progress from distal to proximal body movements, thereby enabling the implementation of symmetric and asymmetric movement sequences.

The barre workout comprised parts 3 and 4. Part 3 consisted of five basic barre movements focused on postural control, lower limb strengthening and mobilization, and dynamic balance, including parallel, first, and second demi plié, relevé, tandu, rond de jambe à terre, and second-position temps lié. After practicing lower-limb movements, the addition of the port de bras enabled systematic coordination training. Adding upper-limb movements also implies an increase in the difficulty level. Owing to joint contractures in the CP, the first foot position was modified from having the heels together to slightly spreading them, and the instructor did not emphasize excessive turnout. In Part 4, the focus is on weight shifting and coordination between the lower and upper limbs through short sequential movements using the barre, which incorporates two or three learned basic barre workouts. This was implemented instead of the center work in a traditional ballet class. We expected that this adaptation would allow the participants to maintain their posture while performing dynamic movements. Participants were encouraged to implement various directions (e.g., front, side, back, and diagonal).

The cool-down consisted of light static stretching of the major muscle groups for 5 min, followed by complete relaxation in the lying position, such as Sava asana in yoga, for 5 min.

### 2.4 Outcome measures

Gait performance was assessed using the 3DGA, which provides a large amount of information, including spatiotemporal gait parameters, dynamic joint motion (kinematics) in three planes (sagittal, frontal, and transverse), and joint movement and power (kinetics) in three planes (25). Outcome measures in our study included spatiotemporal parameters and the GDI score as kinematic parameters. The spatiotemporal parameters include foot-off in gait cycle (%), first double support in gait cycle (%), second double support in gait cycle (%), single support in gait cycle (%), step length (cm), cadence (steps/min), and walking speed (cm/s). The GDI score is calculated based on nine kinematic parameters, including pelvic and hip range of motion in all three planes, knee flexion and extension, ankle dorsiflexion and plantarflexion, and foot progression, as described by Schwartz and Rozumalski (15). It represents a single numerical value ranging from 0 to 100, where a score of 100 or higher indicates the absence of gait pathology, with each 10-point decrease indicating one standard deviation from the mean of healthy controls (15). The GDI score effectively distinguished various levels of gait impairments in adults with spastic CP (22).

The 3DGA was performed 1 week before and 1 week after the intervention. All assessments were conducted at the Laboratory of Motion Analysis Research at Bundang Hospital. The gait analysis is clinically acceptable (26). The outcome measures were collected using a motion capture system (Motion Analyses Corporation, Santa Rosa, CA, USA) equipped with 10 Kestrel digital optical cameras. Reflective markers were placed as per the Helen Hayes marker set by two professional examiners with 15 years of experience in consistent marker placement (27) and national qualifications in motion analysis. The participants walked barefoot on a 10-m walkway six or seven times at a self-selected comfortable speed. Among them, three times that represented their typical gait pattern were selected and averaged to obtain data for the final analysis. All tests were conducted in one session each, both pre and post the intervention, on the day of the examination, with a total measurement time of approximately 20 min.

The feasibility of implementing the intervention was investigated using an exit questionnaire and group interviews conducted after the final intervention (12). The eight items in the exit questionnaire were developed based on Lopez-Ortiz et al.’s study (28) and following the guidelines of a pilot study (12). The questionnaire used a 5-point Likert scale (1 = strongly disagree, 3 = neither agree nor disagree, and 5 = strongly agree). These items were as follows: enjoyment, perceived changes in physical aspects, perceived changes in emotional aspects, perceived appropriateness for people with CP, barriers to participation, usability of the frequency and duration, recommendations to others, and intention to participate again. The interview questions inquired about participants’ opinions on each item. For example, “What aspects did you find enjoyable?”, “What emotional changes did you experience while participating in the ADP?”, “Why would you recommend this activity to others?” Considering the interactivity among the participants, questions were taken into account, allowing for free responses without being strictly bound to them. The interview facilitator created an atmosphere where participants could comfortably and freely express their experiences and opinions. The interview was recorded in its entirety and transcribed by a stenographer. Prior to the group interview, the intention to record, research purpose, confidentiality, and participants’ right to not speak or stop participating at any point were reiterated, and written consent was obtained. The interview response was transcribed, and after analyzing the transcriptions by meaningful units, common themes related to the questions were categorized.

### 2.5 Data analysis

All numerical variables were analyzed using IBM SPSS Statistics for Windows (version 23.0; IBM Corp., Armonk, NY, USA) for data analysis. Descriptive statistics were calculated for all the variables. Differences in each outcome variable pre- and post-intervention were compared using a paired t-test. The exit questionnaire responses were expressed as mean, standard deviation, as well as maximum and minimum scores on a Likert scale. The significance level was set at *p* < 0.05.

## 3 Results

### 3.1 Attendance and demographic characteristics

The demographic characteristics of the participants are presented in Table 1. Initially, 12 participants were recruited. However, after starting the exercise, two individuals dropped out: one at 3 weeks due to inconvenience in accessing the location and another at 7 weeks due to a part-time job. Accordingly, a total of 10 participants completed both assessments and exercises. All 10 participants were female with GMFCS level II, aged 42 to 60 years (mean = 54.1 ± 5.7 years). The height, weight, and BMI showed no differences between pre- and post-assessment. The participants demonstrated an attendance rate of 96.3% over the 24 sessions.

**Table 1:** Demographic characteristics of participants.

Variables	Pre-intervention	Post-intervention	*p*
Age (year)	54.1 ± 5.7		–
Sex	Female		–
GMFCS level	II		–
Type	Spastic diplegic CP		–
Height (cm)	150.65 ± 6.04	150.47 ± 5.87	0.62
Weight (kg)	52.39 ± 8.6	52.1 ± 8.81	0.25
BMI (kg/m^2^)	23.01 ± 3.04	22.93 ± 3.14	0.69
Attendance (%)	96.3		–

Values are mean ± SD. GMFCS, Gross Motor Function Classification System; BMI, Body Mass Index.

### 3.2 Gait

Table 2 presents the changes in the spatiotemporal gait parameters and GDI before and after the intervention. Foot off (*Δ*-0.72%, *p* = 0.47), first double support (Δ-0.2%, *p* = 0.7), second double support (Δ1.5%, *p* = 0.43), and single support (Δ0.64%, *p* = 0.46) presented no significant difference. Step length (Δ1.48 cm, *p* = 0.28), cadence (Δ3.95 steps/min, *p* = 0.24), and walking speed (Δ6.41 cm/s, *p* = 0.11) showed an increasing tendency but no statistically significant difference. However, statistically significant differences were observed on GDI after intervention (Δ5.74 points, *p* = 0.02), showing a large effect size (*d* = 0.76) (29).

**Table 2:** Changes of the spatiotemporal gait parameters and Gait Deviation Index (GDI) for pre- and post-adapted Dance Program for adults with spastic diplegic cerebral palsy.

Outcome measure	Pre-intervention	Post-intervention	*t*	*p*	ES*(d)*
Foot off (%)	60.87 ± 3.65	60.15 ± 2.28	0.75	0.47	0.33
First Double Support (%)	9.59 ± 2.61	9.39 ± 2.81	0.39	0.70	0.1
Second Double Support (%)	10.9 ± 2.99	9.74 ± 3.68	0.83	0.43	0.49
Single Support (%)	40.37 ± 2.6	41.01 ± 2.6	−0.77	0.46	0.35
Step Length (cm)	51.74 ± 9.91	53.22 ± 9.61	−1.16	0.28	0.25
Cadence (steps/min)	114.51 ± 13.5	118.46 ± 12.6	−1.25	0.24	0.43
Walking Speed (cm/s)	99.27 ± 22.27	105.68 ± 24.65	−1.75	0.11	0.39
GDI (point)	84.75 ± 9.99	90.49 ± 11.4	−3.02	0.02	0.76

Values are mean ± SD. ES, effect size.

### 3.3 Feasibility

Table 3 presents participants’ responses to the exit questionnaire and comments from the group interview. Enjoyment showed 4.7 ± 0.46 points. Participants mentioned the role of music in making exercise more enjoyable and less tedious, providing a refreshing alternative to traditional therapy or exercise. Several participants expressed pride in their ability to dance, despite initial doubts about its feasibility for individuals with CP. Perceived changes in physical aspects presented 4.6 ± 0.49 points. The majority of participants attributed dynamic movements using the bar to improvements in left–right alignment, posture adjustment, and increased leg strength. Participants reported feeling a noticeable increase in leg strength, with one individual noting, “I feel like there is more power in my legs.” Another thing is that looking in the mirror was effective in correcting their posture. Perceived changes in emotional aspects (mean = 3.9 ± 0.74 points). Participants noted increased confidence as their posture improved and their gait became more dynamic, leading to a more vibrant daily life. Participants also appreciated the opportunity to share enjoyable experiences with others, enhancing camaraderie. Perceived appropriateness for people with CP showed 4.7 ± 0.46 points. Participants acknowledged the effectiveness of incorporating floor and barre workouts, as well as using the bar, in enabling them to perform more dynamic movements while ensuring safety. Barriers to participation exhibited 1.2 ± 0.42 points. In addition to one participant dropping out due to difficulty accessing the intervention location, others mentioned the inconvenience of using transportation services (such as disability call taxis) to get to the class location. The usability of the frequency and duration presented 4.6 ± 0.66 points. There was high satisfaction with the 90-min class held twice a week, compared to other classes typically lasting 50 min once a week. Instructors noted that due to slower movement transitions and progression in individuals with CP, securing sufficient time is essential for effective intervention implementation. Regarding recommendations to others (mean = 4.6 ± 0.52 points) and participation again (mean = 4.8 ± 0.42 points), participants expressed a desire to participate again and a willingness to recommend the class to others, suggesting starting at a slightly younger age.

**Table 3:** Results from the exit questionnaire and group interview.

Questions	Mean ± SD, point (Min, Max)	Main comments for the questions
Enjoyment	4.7 ± 0.46 (4, 5)	Using music, engaging in dance, a different experience from typical exercise or physical therapy
Perceived changes in physical aspects	4.6 ± 0.49 (4, 5)	Left–right alignment, control posture, energetic walking, posture straightening, a slightly softened movement
Perceived changes in emotional aspects	3.9 ± 0.74 (3.25, 4)	Confidence derived from adjusted posture, a lively life, and the joy of being together
Perceived appropriateness for people with CP	4.7 ± 0.46 (4,5)	Effectiveness of using bar, combine bar work and floor work
Barriers to participation	1.2 ± 0.42 (1, 1)	Location
Usability of the frequency and duration	4.6 ± 0.66 (3, 5)	Better than the typical duration of 50 min
Recommendations to others	4.8 ± 0.42 (4, 5)	Starting exercise at a younger age
Intention to participate again	4.7 ± 0.46 (4, 5)	–

## 4 Discussion

This pilot study aimed to investigate the effects of ADP on gait in adults with spastic diplegic CP (GMFCS level II). The ADP comprised a structured dance program consisting of floor and barre workouts. The intervention was conducted twice a week for 90 min each session, for a total of 24 sessions. Participants attended 96.3% of the sessions. While the step length, cadence, and gait speed increased, no statistically significant differences were observed. However, in terms of kinematic variables, there was a significant increase of 5.74 points in the GDI, demonstrating a large effect size (*d* = 0.76), indicating the benefits of ADP in improving gait patterns in adults with spastic diplegic CP. This result can be discussed in terms of the following two attributes.

First, music in dance interventions plays a crucial role in synchronizing rhythmic signals and motor responses in the rehabilitation of movement disorders (30). Previous studies (10, 31, 32) reporting improvements in gait performance among neurorehabilitation patients after dance interventions have emphasized the rhythmic auditory stimulation theory (RAS) as a basis for functional improvement. Kim et al. (33) conducted a intervention using keyboards (30-min individual sessions three times per week for 4 weeks) for adults with CP and reported improvements in cadence, velocity, stride length, and kinematic parameters following the intervention. Shin et al. (34) reported a GDI increase of 3.48 points in hemiplegic adults with either cerebral palsy or stroke after gait training based on RAS treatment (30 min, three times per week for 4 weeks). Moreover, in RAS, a significant improvement of 3.9 points in GDI was observed, while the Bobath treatment showed a decrease of −1.3 points (35). In our study, participants were encouraged to move in sync with the rhythm of ballet music during the barre workout, whereas in the floor workout, either a simple beat was provided or instructors offered verbal counting cues to train participants to control their movements according to the given tempo. In line with this, our findings are consistent with previous studies reporting the effects of rhythmic interventions.

In addition, ADP involves multisensory activity that requires the body to move while simultaneously processing visual, tactile, and auditory stimuli (5). Rehfeld et al. (31) reported improvements in motor and proprioceptive functions after dance training. They noted that the complexity of dance movements not only enhances awareness of the body’s position in space and body segmentation but also improves coordination by engaging multiple body parts simultaneously. In our study, during the floor workout, participants initially focused on enhancing proprioception for the movements of the upper and lower limbs separately. This progressed to patterns of whole-body coordination incorporating symmetric movements (e.g., moving the same-side arm and leg) and asymmetric movements (e.g., moving the opposite arm and leg). During the barre workout, the participants performed a series of tasks that included recognizing body movements, adjusting posture in space, imitating movements, and learning new movements. Consequently, the improved GDI scores observed in this study may be attributed to several elements integrated into the ADP, including enhanced proprioception, increased awareness of body positional changes in space, and better overall body coordination. Lakes et al. (17) described weight shifting as a key factor in improving gait speed, stride length, and selective voluntary motor control, providing a different pattern of improvement in the hip and subtalar joints after a ballet intervention.

### 4.1 Feasibility

With regard to the feasibility of ADP, participants perceived dance as a different form of activity from traditional exercises or therapies, showing high satisfaction with dancing to music. There were no relevant accidents or harm in implementing the ADP. While dance is increasingly used in therapeutic settings (5, 9), there is still a tendency for people to perceive it as a professional domain, similar to dance performed by people without disabilities. It is necessary to lower barriers and expand the perception that “everybody can dance.” Regarding physical changes, the participants expressed high satisfaction with their adjusted posture and energetic walking while performing the ADP. In line with studies indicating a significant relationship between physical health, social participation, and emotional health (32, 36), it has been observed that emotional changes stem from physical changes in the present study. Social participation has been highlighted as a benefit of dance, emphasizing its role in fostering social engagement (7). The participants in this study also expressed the joy of being together. This study shows that while social participation through dance does not involve community events or performances (9), participating in group classes can be a form of social engagement, allowing individuals to meet others and share experiences. Considering these factors, additional dance classes should be offered. However, as noted as a barrier in this study and by Lopez-Ortiz et al. (28), accessibility between homes and class locations makes it difficult to sustain consistent class attendance. Accessibility to transportation is crucial when considering interventions for individuals with disabilities.

**Figure 2:**
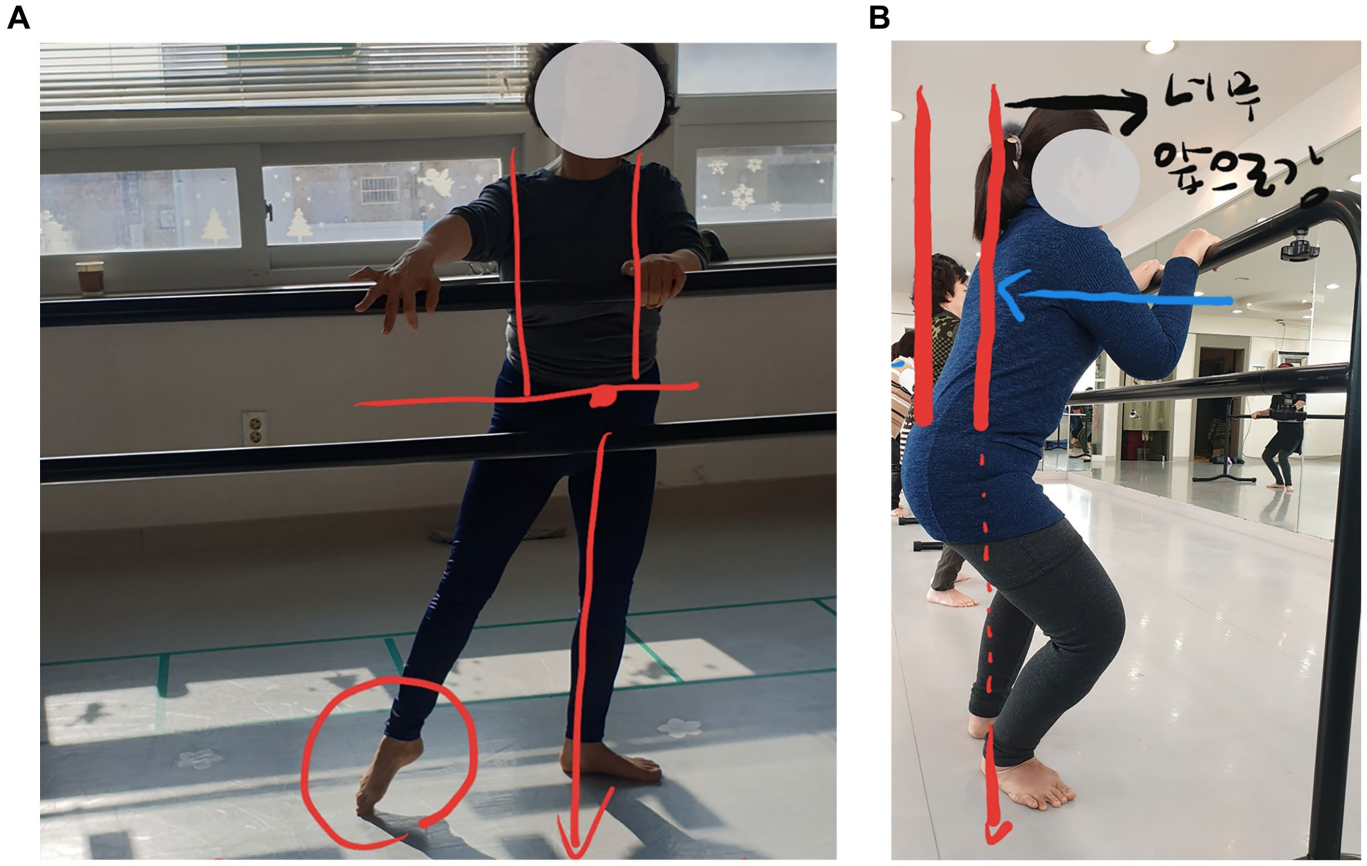
Examples of feedback on posture alignment using photographs (Photo reproduced with permission) **(A)** Demi plié posture alignment, **(B)** Alignment during weight shifting.

In terms of appropriateness, conducting floor and barre workouts together in one class, along with the use of bars and mirrors, was suitable for a dance program for people with CP. There was no perception that the combination of floor and barre workouts in one class was burdensome or risky. Instead, they were satisfied after engaging in sufficient stretching and slow whole-body coordination training during floor workouts and then processing dynamic movements during barre workouts. Moreover, the participants were highly satisfied with the 90-min sessions. In particular, the instructors pointed out that people with CP require a slower pace of movement, sufficient time for transitions between movements, and consideration of rest periods during class. Given the actual training duration, we suggest that a 90-min class per session is appropriate. We installed the bar horizontally instead of vertically, as is typically done in general ballet classes. This was modified for individuals with CP. Participants held the bar with both hands, enabling safe and easy weight shifting from side to side while facing the mirror, which helped them align their postures. The participants reported that supporting the bar with both hands helped them balance and shift their weight more effectively. Additionally, feedback from instructors on posture seemed to influence participants’ self-correction of posture, indicating the importance of providing verbal and visual feedback during program implementation (Figure 2).

The participants expressed a willingness to recommend this class to others, suggesting that individuals should participate in such classes for posture correction at a younger age. This finding is consistent with those of previous studies that showed deterioration in physical function in the late twenties and thirties (4, 37). Based on these results, the necessity for early intervention in adults with CP should be emphasized.

Our results support the feasibility of the ADP for improving gait in adults with spastic diplegic CP. However, the lack of a control group, non-random sampling, absence of follow-up, and the fact that all participants were female limit the generalizability of the research findings. Despite these limitations, this study provides meaningful information regarding the implementation of dance to guide further research and large-scale studies. First, it provides valuable results by examining the impact of the ADP on gait in adults with spastic diplegic CP, a parameter that has not been previously explored in dance studies involving adult with CP. Second, by assessing the feasibility of various aspects of the research from the participants’ perspective, researchers can refine their approach, mitigate potential challenges, and successfully conduct larger studies. Third, we enhanced the reproducibility of the study by providing sufficient details regarding program implementation.

## 5 Conclusion

In conclusion, ADP consisting of floor and barre workouts can be an effective intervention for enhancing gait patterns in adults with spastic diplegic CP. The participants expressed high levels of emotional and physical satisfaction. The use of bars and mirrors was found to be effective in posture control and safe weight shifting, and sufficient session duration (e.g., 90 min) was deemed suitable for dance classes for people with CP. Additionally, starting treatment at a younger age was recommended. This study suggests further randomized controlled trials to provide strong evidence of the effect of dance interventions on gait function in adults with CP.

## Data Availability

The original contributions presented in the study are included in the article/Supplementary material, further inquiries can be directed to the corresponding author.

## References

[1] Strauss D Ojdana K Shavelle R Rosenbloom L. Decline in function and life expectancy of older persons with cerebral palsy. Neurorehabil Neural Repair. (2004) 19:69–78. doi: 10.3233/NRE-2004-19108, PMID: 14988589

[2] Peterson MD Ryan JM Hurvitz EA Mahmoudi E. Chronic conditions in adults with cerebral palsy. JAMA. (2015) 314:2303–5. doi: 10.1001/jama.2015.11025 26624831 PMC4862577

[3] Andersson C Mattsson E. Adults with cerebral palsy: a survey describing problems, needs, and resources, with special emphasis on locomotion. Dev Med Child Neurol. (2007) 43:76–82. doi: 10.1111/j.1469-8749.2001.tb00719.x, PMID: 11221908

[4] Jahnsen R Villien L Egeland T Stanghelle JK Holm I. Locomotion skills in adults with cerebral palsy. Clin Rehabil. (2004) 18:309–16. doi: 10.1191/0269215504cr735oa 15137562

[5] Zuliani FG Fonseca BHS Miranda JMA Sande de Souza LAP Luvizutto GJ. A scoping systematic review of dance application as a rehabilitation tool in adults and older individuals with neurological diseases. Phys Ther Rev. (2023) 28:291–302. doi: 10.1080/10833196.2023.2276971

[6] Dhami P Moreno S DeSouza JF. New framework for rehabilitation–fusion of cognitive and physical rehabilitation: the hope for dancing. Front Psychol. (2015) 5:1478. doi: 10.3389/fpsyg.2014.01478 25674066 PMC4309167

[7] Lopez-Ortiz C Gaebler-Spira DJ McKeeman SN McNish RN Green D. Dance and rehabilitation in cerebral palsy: a systematic search and review. Dev Med Child Neurol. (2019) 61:393–8. doi: 10.1111/dmcn.14064, PMID: 30350851

[8] Cherriere C Robert M Fung K Tremblay Racine F Tallet J Lemay M. Is there evidence of benefits associated with dancing in children and adults with cerebral palsy? A scoping review. Disabil Rehabil. (2020) 42:3395–402. doi: 10.1080/09638288.2019.1590866, PMID: 30973761

[9] Duarte Machado E Cole MH Miller L McGuckian TB Wilson PH. The efficacy of dance interventions for the activity and participation of individuals with cerebral palsy - a systematic review and meta-analysis. Disabil Rehabil. (2024) 46:1485–501. doi: 10.1080/09638288.2023.2200259, PMID: 37122166

[10] Joung HJ Yang HK Lee Y. Effect of dance on balance, mobility, and activities of daily living in adults with cerebral palsy: a pilot study. Front Neurol. (2021) 12:663060. doi: 10.3389/fneur.2021.663060, PMID: 34025566 PMC8137835

[11] Terada K Satonaka A Terada Y Suzuki N. Training effects of wheelchair dance on aerobic fitness in bedridden individuals with severe athetospastic cerebral palsy rated to GMFCS level V. Eur J Phys Rehabil Med. (2017) 53:744–50. doi: 10.23736/S1973-9087.17.04486-0, PMID: 28178772

[12] Teresi JA Yu X Stewart AL Hays RD. Guidelines for designing and evaluating feasibility pilot studies. Med Care. (2022) 60:95–103. doi: 10.1097/MLR.0000000000001664, PMID: 34812790 PMC8849521

[13] Ries AJ Novacheck TF Schwartz MH. The efficacy of ankle-foot orthoses on improving the gait of children with diplegic cerebral palsy: a multiple outcome analysis. PM R. (2015) 7:922–9. doi: 10.1016/j.pmrj.2015.03.005, PMID: 25771349

[14] Massaad A Assi A Skalli W Ghanem I. Repeatability and validation of gait deviation index in children_ typically developing and cerebral palsy. Gait Posture. (2014) 39:354–8. doi: 10.1016/j.gaitpost.2013.08.001, PMID: 24079975

[15] Schwartz MH Rozumalski A. The gait deviation index: a new comprehensive index of gait pathology. Gait Posture. (2008) 28:351–7. doi: 10.1016/j.gaitpost.2008.05.001, PMID: 18565753

[16] Hoffmann TC Glasziou PP Boutron I Milne R Perera R Moher D. Better reporting of interventions: template for intervention description and replication (TIDieR) checklist and guide. BMJ. (2014) 348:g1687. doi: 10.1136/bmj.g1687 24609605

[17] Lakes KD Sharp K Grant-Beuttler M Neville R Haddad F Sunico R. A six week therapeutic ballet intervention improved gait and inhibitory control in children with cerebral palsy-a pilot study. Front Public Health. (2019) 7:137. doi: 10.3389/fpubh.2019.00137, PMID: 31294009 PMC6603155

[18] Yi YG Jung SH Bang MS. Emerging issues in cerebral palsy associated with aging: a physiatrist perspective. Ann Rehabil Med. (2019) 43:241–9. doi: 10.5535/arm.2019.43.3.241, PMID: 31311245 PMC6637058

[19] Palisano R Rosenbaum P Walter S Russell D Wood E Galuppi B. Development and reliability of a system to classify gross motor function in children with cerebral palsy. Dev Med Child Neurol. (1997) 39:214–23. doi: 10.1111/j.1469-8749.1997.tb07414.x 9183258

[20] Jahnsen R Aamodt G Rosenbaum P. Gross motor function classification system used in adults with cerebral palsy: agreement of self-reported versus professional rating. Dev Med Child Neurol. (2006) 48:734–8. doi: 10.1017/S0012162206001575, PMID: 16904019

[21] Kinsner-Ovaskainen A. Lanzoni M. Delobel M. Ehlinger V. Arnaud C. Martin S. Surveillance of cerebral palsy in Europe: Development of the JRC-SCPE Central Database and Public Health Indicators, EUR 28935 EN. Luxembourg: Publications Office of the European Union. (2017).

[22] Maanum G Jahnsen R Stanghelle JK Sandvik L Larsen KL Keller A. Face and construct validity of the gait deviation index in adults with spastic cerebral palsy. J Rehabil Med. (2012) 44:272–5. doi: 10.2340/16501977-0930, PMID: 22214985

[23] Toldi J Escobar J Brown A. Cerebral palsy: sport and exercise considerations. Curr Sports Med Rep. (2021) 20:19–25. doi: 10.1249/JSR.0000000000000798, PMID: 33395127

[24] Scheidler AM Kinnett-Hopkins D Learmonth YC Motl R Lopez-Ortiz C. Targeted ballet program mitigates ataxia and improves balance in females with mild-to-moderate multiple sclerosis. PLoS One. (2018) 13:e0205382. doi: 10.1371/journal.pone.0205382, PMID: 30335774 PMC6193654

[25] Min JJ Kwon SS Sung KH Lee KM Chung CY Park MS. Factors affecting GDI improvement after single event multilevel surgery in patients with cerebral palsy. Gait Posture. (2020) 80:101–5. doi: 10.1016/j.gaitpost.2020.05.033, PMID: 32497978

[26] McGinley JL Baker R Wolfe R Morris ME. The reliability of three-dimensional kinematic gait measurements: a systematic review. Gait Posture. (2009) 29:360–9. doi: 10.1016/j.gaitpost.2008.09.003 19013070

[27] Kadaba MP Ramakrishnan HK Wootten ME. Measurement of lower extremity kinematics during level walking. J Orthop Res. (1990) 8:383–92. doi: 10.1002/jor.1100080310 2324857

[28] Lopez-Ortiz C Gladden K Deon L Schmidt J Girolami G Gaebler-Spira D. Dance program for physical rehabilitation and participation in children with cerebral palsy. Arts Health. (2012) 4:39–54. doi: 10.1080/17533015.2011.564193, PMID: 25431617 PMC4226305

[29] Cohen L. Statistical power analysis for the behavioral sciences. 2nd ed. New York: Routledge (2013).

[30] Thaut MH Abiru M. Rhythmic auditory stimulation in rehabilitation of movement disorders: a review of current research. Music Percept. (2010) 27:263–9. doi: 10.1525/mp.2010.27.4.263

[31] Rehfeld K Luders A Hokelmann A Lessmann V Kaufmann J Brigadski T. Dance training is superior to repetitive physical exercise in inducing brain plasticity in the elderly. PLoS One. (2018) 13:e0196636. doi: 10.1371/journal.pone.0196636, PMID: 29995884 PMC6040685

[32] Bjornson KF Belza B Kartin D Logsdon R McLaughlin J Thompson EA. The relationship of physical activity to health status and quality of life in cerebral palsy. Pediatr Phys Ther. (2008) 20:247–53. doi: 10.1097/PEP.0b013e318181a959, PMID: 18703962 PMC3644992

[33] Kim SJ Yoo GE Shin YK Cho SR. Gait training for adults with cerebral palsy following harmonic modification in rhythmic auditory stimulation. Ann N Y Acad Sci. (2020) 1473:11–9. doi: 10.1111/nyas.14306, PMID: 32356332

[34] Shin YK Chong HJ Kim SJ Cho SR. Effect of rhythmic auditory stimulation on hemiplegic gait patterns. Yonsei Med J. (2015) 56:1703–13. doi: 10.3349/ymj.2015.56.6.1703, PMID: 26446657 PMC4630063

[35] Kim SJ Kwak EE Park ES Cho SR. Differential effects of rhythmic auditory stimulation and neurodevelopmental treatment/Bobath on gait patterns in adults with cerebral palsy: a randomized controlled trial. Clin Rehabil. (2012) 26:904–14. doi: 10.1177/0269215511434648 22308559

[36] Gaskin CJ Morris T. Physical activity, health-related quality of life, and psychosocial functioning of adults with cerebral palsy. J Phys Act Health. (2008) 5:146–57. doi: 10.1123/jpah.5.1.146, PMID: 18209260

[37] Usuba K Oddson B Gauthier A Young NL. Changes in gross motor function and health-related quality of life in adults with cerebral palsy: an 8-year follow-up study. Arch Phys Med Rehabil. (2014) 95:2071–2077.e1. doi: 10.1016/j.apmr.2014.05.018 24909589

