# Supplementary material for: Effect of adapted dance program on gait in adults with cerebral palsy: a pilot study
Source: Front Neurol. 2024 Oct 14;15:1443400. doi: 10.3389/fneur.2024.1443400 (PMC11513902; doi:10.3389/fneur.2024.1443400)
Supplement: Supplementary file 1 [file Data_Sheet_1.PDF]

Supplementary Material

A list of detailed movement descriptions in the Adapted Dance Program (ADP) for cerebral palsy

- **Introduction**

The ADP consists of floor and barre workout. The floor workout is performed in a lying position to mitigate tension and compensatory movement patterns. This focuses on stretching, increasing range of motion (ROM), body perception, and coordination between the upper and lower limbs. In particular, works 5 and 9 are based on Bartenieff Fundamentals (BF) basic movements. The barre workout is performed in a standing position using a horizontal bar for safety. This focuses on postural control, dynamic balance, strengthening, and coordination. It allows for movement sequence while maintaining proper postural alignment.

- **Suggestion**

. After practicing lower limb movement, add port de bras.
. Do not demand excessive turnout.
. The first feet position is adjusted so that the heels are slightly apart, rather than following the traditional posture of keeping the heels together.
. Provide feedback on alignment and elongation of the spine so that participants can maintain proper posture. Use friendly imagery (e.g., flowing water, their own postural picture) to explain achieving dynamic alignment.

• **Movement technique**

**Floor workout**	
**Work 1. Big X full body stretching** (4 reps in each direction, 2 sets)	**Work 2. Lying feet point and flex** (8 reps, 3 sets)
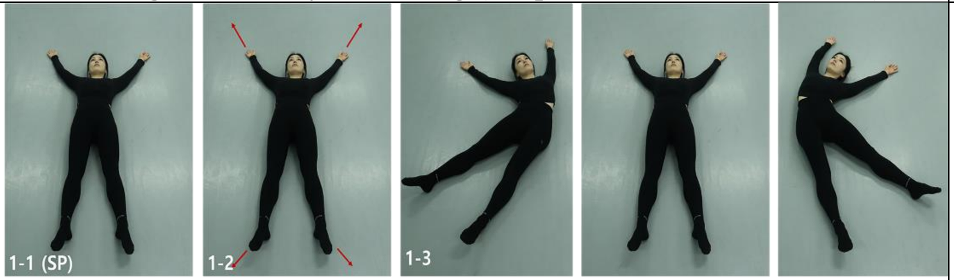	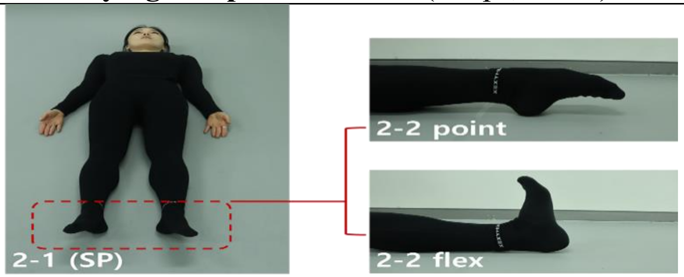
1-1. (SP) Lie down in a big X<br>1-2. Elongate each arm upward and leg downward separately<br>1-3. Make a semicircle with your body to the right and left	2-1. (SP) Lie down on the ground<br>2-2. Point and flex your feet
**Work 3. Hamstring stretching** (4 reps, 2 sets)	**Work 4. Hip joint ab and ad** (8 reap, 2 sets)
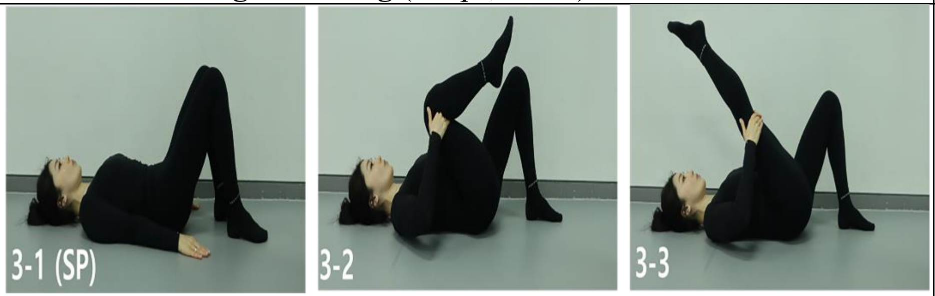	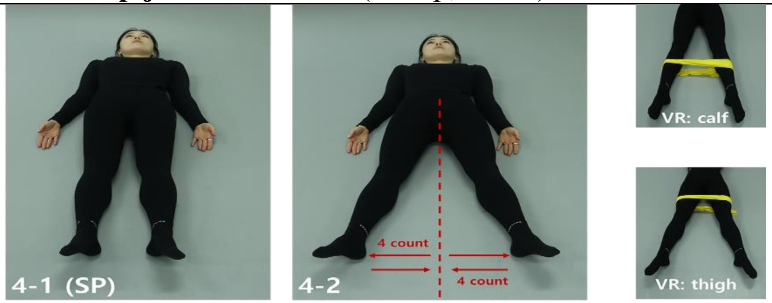
3-1. (SP) supine position<br>3-2. Grab one thigh with both hands.<br>3-3. Straighten your knee as much as possible and hold for 20 seconds.	4-1 (SP) Lie down with both legs together<br>4-2. (4 counts) Abduct the hip joint, (4 counts) Adduct the hip joint<br>(VR) Using thera-bend on calf or thigh

**Work 5. Knee drop** (4 reps in each step, 2 sets)	**Work 6. Toe tapping** (8 reps, 2 sets)
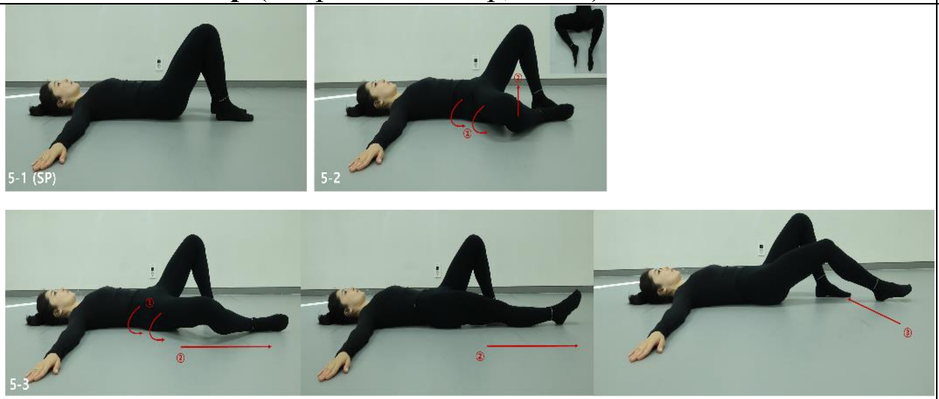	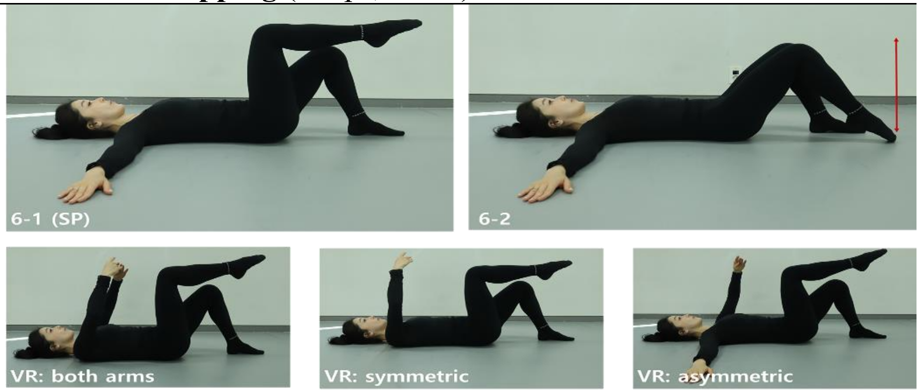
5-1. (SP) supine position<br>5-2. Gently lower one knee to the side, then return to the SP<br>5-3. Lower one knee, extend it downwards, then return to the SP	6-1. (SP) Lift one bent knee to a 90-degree angle<br>6-2. Lightly tap the floor with the tip of your foot, then return to the SP<br>(VA) arm movement (up & down) and toe tap simultaneously
**Work 7. Lying first position plié** (8 reps, 2 sets)	**Work 8. Lying port de bra** (8 reps in each step, 2 sets)
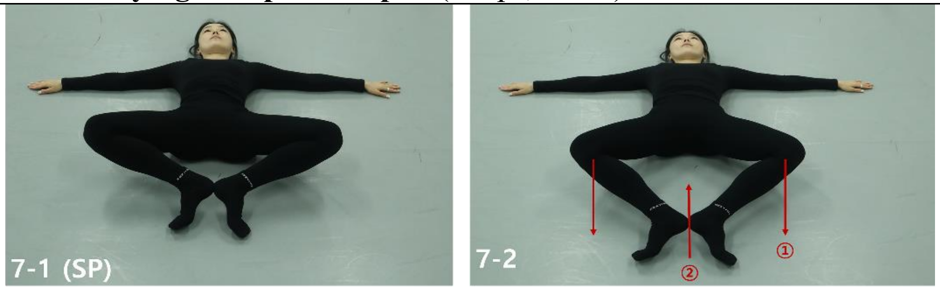	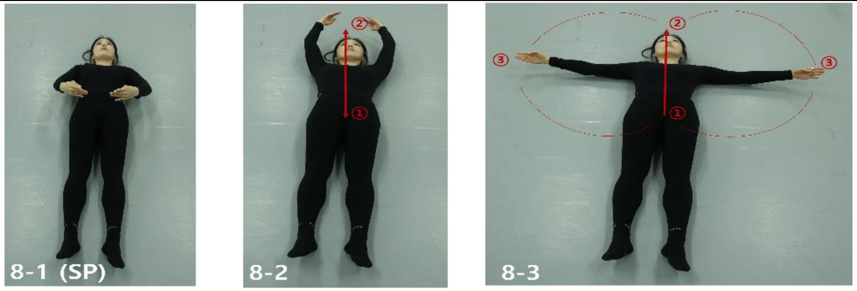
7-1. (SP) Lie down for the first plié<br>7-2. (4 counts) Gently extend the legs downward, (4 counts) slowly bend knee and return to the SP	8-1. (SP) Lie down in the 1st arm position<br>8-2. Repeat 1st → 5th arm positions, and return to the SP<br>8-3. Repeat 1st → 5th →2nd arm positions, and return to the SP

Work 9. Upper lower limbs connectivity sequence (4 reps in each step, 2 sets)

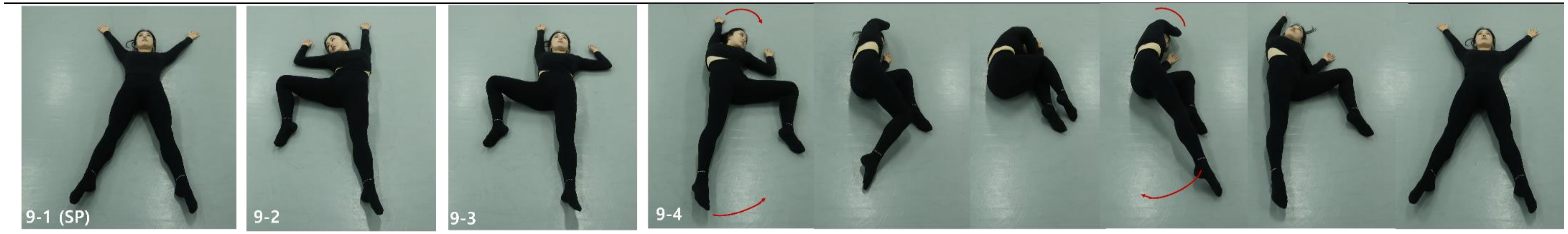

9-1. (SP) Lie down in a big X
9-2. (4 counts, symmetric) On the same side, bend the knee and arm simultaneously while tilting the trunk, slowly return to the SP
9-3. (4 counts, asymmetric) Bend the knee and the arm on the opposite side simultaneously, while keeping the other arm and leg stretched
9-4. (8 counts) perform 9-2, the opposite arm and leg rotate to the moving side, make a baby pose, (8 counts) return to the SP

Barre workout

Work 10. Demi plié (4 reps with each position, 2 sets)

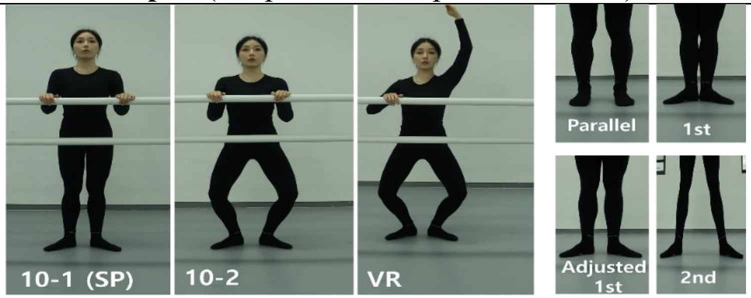

10-1. (SP) Standing position in the parallel, 1st, adjusted 1st, 2nd feet position ** If the 1st feet position is difficult, perform an adjusted 1st.
10-2. (4 counts) slowly bend knee, (4 counts) straighten knees
(VR) add arm movement

Work 11. Relevé (4 reps in each step, 2 sets)

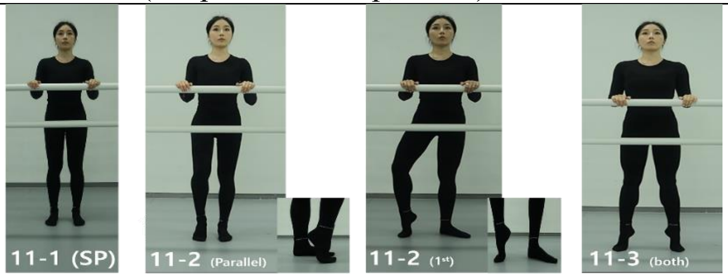

11-1. (SP) Standing in the parallel and 1st feet position
11-2. (2 counts) lift a heel with a bent knee and return to the SP
11-3. (2 counts) lift both heels with straight knees and return to the SP.

<table>
<tr><td>Work 12. Tandu (4 reps with each direction, 2 sets)</td><td>Work 13. Rond de jambe à terre (4 reps with each step, 2 sets)</td></tr>
<tr><td>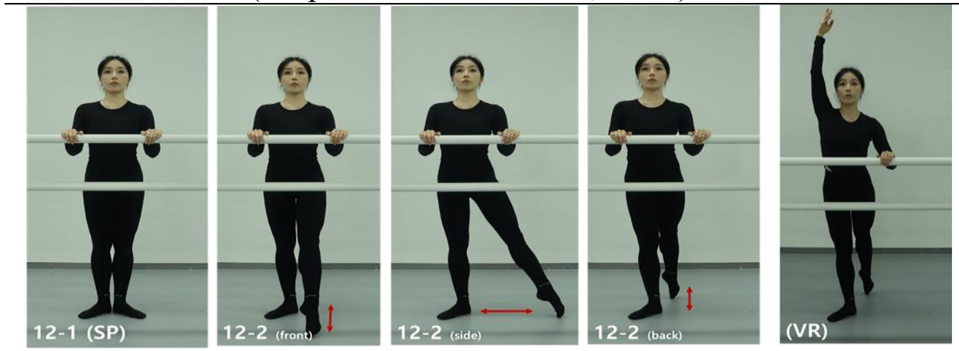
</td><td>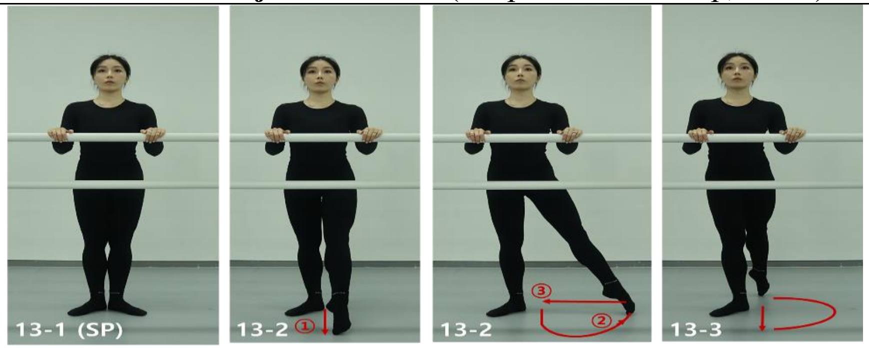
</td></tr>
<tr><td>12-1. (SP) Standing in 1ST feet position
12-2. Lightly slide your toes on the ground in each direction (front, side, back) while stretching your knee, then return to the SP
(VR) add arm movement</td><td>13-1. (SP) Standing in 1ST feet position
13-2. Extend the leg to the front, make a quarter circle with the toes on the ground, then return to the SP
13-3. Extend the leg to the front, make a half circle with the toes on the ground, then return to the SP</td></tr>
<tr><td colspan="2">Work 14. Second position temps lié and diagonal variation (4 reps on each step, 2 sets)</td></tr>
<tr><td colspan="2">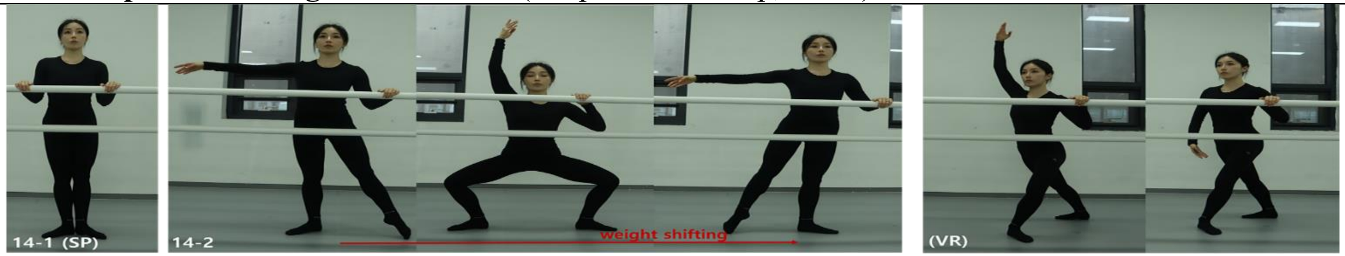
</td></tr>
<tr><td colspan="2">14-1. (SP) Standing in 1ST feet position
14-2. Extend the leg to the side, perform 2nd plié, straighten the knee while shifting weight to the other side, then return to the SP.
(VR) Add the diagonal position: Perform (14-2), then extend the leg diagonally backward, repeat the arm movement up and down, return to the SP</td></tr>
</table>

SP: Starting Position; VR: Variation; REPS: repeats. Photo reproduced with permission.
